# Comparison of high-flow nasal oxygen therapy and noninvasive ventilation in suspected sepsis patients with acute respiratory distress in the emergency department: a retrospective cohort study

**DOI:** 10.1186/s12245-025-00842-2

**Published:** 2025-03-10

**Authors:** Chuenruthai Angkoontassaneeyarat, Prapimporn Charoenphon, Pitsucha Sanguanwit, Chaiyaporn Yuksen, Suteenun Seesuklom

**Affiliations:** Department of Emergency Medicine, Faculty of Medicine, Ramathibodi Hospital, Mahidol University, 270 Rama VI Road, Thung Phaya Thai, Ratchathewi, Bangkok, 10400 Thailand

**Keywords:** Sepsis, Respiratory distress, High-flow nasal cannula, Noninvasive ventilation, Intubation

## Abstract

**Introduction:**

High-flow nasal cannula (HFNC) and non-invasive ventilation (NIV) are widely utilized respiratory support modalities for patients presenting with suspected sepsis and respiratory distress.This study aims to compare the 48-hour intubation rates between HFNC and NIV therapies in patients with suspected sepsis and respiratory distress.

**Methods:**

This retrospective cohort study collected data over a 2-year period (January 2022 to December 2023) from patients presenting to the ED of Ramathibodi Hospital with suspected sepsis who received respiratory support with either HFNC or NIV. To analyze the incidence of intubation and 28-day mortality, we employed multivariable Cox regression to estimate hazard ratios (HRs). The hospital length of stay and ventilator-free days at 28 days were compared using Gaussian regression analysis.

**Results:**

A total of 546 patients met the inclusion criteria. The intubation at 48 h was 17.39% in the HFNC group and 19.47% in the NIV group (adjusted HR 0.74; 95% CI, 0.48 to 1.15; *p* = 0.18). The HFNC group demonstrated a trend toward lower 28-day mortality than the NIV group (adjusted HR 0.34; 95% CI, 0.12 to 1.02; *p* = 0.053). Additionally, the HFNC group had significantly more ventilator-free days (adjusted mean difference 1.46 days; 95% CI, 0.11 to 2.80; *p* = 0.034).

**Conclusions:**

In patients with suspected sepsis and acute respiratory distress, HFNC therapy did not significantly reduce the 48-hour intubation compared to NIV. However, HFNC was associated with a trend toward lower 28-day mortality and a significantly greater number of ventilator-free days on day 28.

**Trial registration:**

This trial was retrospectively registered in the Thai Clinical Trial Registry on 09 November 2023. The TCTR identification number is TCTR20231109004.

**Supplementary information:**

The online version contains supplementary material available at 10.1186/s12245-025-00842-2.

## Introduction

Sepsis is a highly prevalent and life-threatening condition commonly encountered in the emergency department (ED). It represents a systemic response to infection that results in acute organ dysfunction. One of the severe complications of sepsis is acute respiratory failure (ARF), which is particularly prevalent in critically ill patients [1]. Approximately 80% of lung injuries in intensive care units (ICUs) occur in the context of sepsis, with over 60% of patients with ARF requiring intubation [2, 3]. Sepsis impacts pulmonary function through multiple mechanisms, including systemic inflammation that leads to pulmonary endothelial damage and microcirculatory dysfunction [4–6]. ARF is a critical complication of sepsis, and sepsis-induced acute respiratory distress syndrome (ARDS) is associated with significantly elevated morbidity and mortality. Patients with sepsis-induced ARDS face a fourfold increased risk of in-hospital mortality [7].

Noninvasive ventilation (NIV) has been widely utilized as an alternative respiratory support strategy to minimize the need for invasive ventilation in various clinical conditions [8–10]. Although NIV offers comparable physiological benefits, including improved gas exchange and reduced respiratory effort, its application may be limited in patients with hemodynamic instability. Furthermore, current evidence remains insufficient to recommend NIV over invasive ventilation for managing hypoxemic respiratory failure caused by sepsis [11].

The high-flow nasal cannula (HFNC), a recently developed noninvasive oxygenation device, has emerged as a potential alternative to NIV for patients with ARF [11–13]. Studies involving patients with acute hypoxemic respiratory failure have reported no significant differences in intubation between those treated with HFNC and those managed with NIV [14, 15]. Moreover, a randomized trial conducted on a large cohort of patients presenting to the ED with ARF found that HFNC was not inferior to NIV regarding intubation outcomes [16].

However, there is limited research directly comparing the use of HFNC and NIV in the management of sepsis. Although the 2021 Surviving Sepsis Campaign guidelines recommend HFNC over NIV for sepsis-related hypoxemic respiratory failure, the supporting evidence is of low quality [11].

To address this knowledge gap, we conducted a retrospective study of patients presenting to the ED with suspected sepsis and acute respiratory distress. This study aimed to evaluate whether HFNC or NIV could reduce the need for endotracheal intubation and improve clinical outcomes. The primary objective of this study was to compare the incident of 48-hour intubation between HFNC and NIV in patients with suspected sepsis and respiratory distress in the ED. Secondary objectives included comparing the 28-day mortality, the length of hospital stays, and the number of ventilator-free days within 28 days between the two treatment modalities.

## Methods

### Study design and setting

We conducted a therapeutic retrospective cohort study with an explanatory model design in the ED of Ramathibodi Hospital, a university-affiliated tertiary care hospital in Bangkok, Thailand. The data for this study were collected over a two-year period, spanning from January 1, 2022, to December 31, 2023. NIV has been utilized in the ED since 2006, initially for the management of acute heart failure, with subsequent expanded indications, including acute respiratory distress [10]. HFNC therapy was introduced during the COVID-19 pandemic and has remained widely used in ED.

Patient data for cases of suspected sepsis and acute respiratory distress were retrieved from the Ramathibodi electronic medical record database (RAMA-EMR) for the period between January 1, 2022, and December 31, 2023. Informed consent was waived, as the data were collected retrospectively and anonymized. The study protocol was approved by the Human Research Ethics Committee, Faculty of Medicine, Ramathibodi Hospital, Mahidol University (IRB COA. MURA2023/16).

### Participants

We enrolled patients who presented to the ED of Ramathibodi Hospital with suspected sepsis and were managed according to the Ramathibodi sepsis protocol. Patients were eligible for inclusion if they were described as having suspected sepsis with acute respiratory distress and met all of the following criteria: Age ≥ 18 years and suspected sepsis, as determined by a physician based on clinical suspicion of infection from history or physical examination, along with at least one of the following indicators: Quick Sequential Organ Failure Assessment (qSOFA) score ≥ 2 [1], Ramathibodi Early Warning Score (REWs) ≥ 4 [17] or clinical signs of respiratory distress, including any of the following: use of accessory muscles, abdominal paradox, respiratory rate ≥ 25 breaths per minute, oxygen saturation ≤ 90%, or a PaO2-to-FiO2 ratio ≤ 300 [14, 18, 19], (Supplement 2).

We excluded patients who were treated in outpatient departments or by emergency medical services (EMS) prior to transfer to the ED and those referred from other hospitals. Additional exclusion criteria included patients who switched between HFNC and NIV therapies in the ED, those on home-positive airway pressure therapy, individuals with do-not-resuscitate (DNR) orders, and patients with incomplete data in the RAMA-EMR.

### Respiratory support

The attending physician determines the selection between HFNC and NIV depending on factors such as underlying diseases, symptoms, and secretion clearance for patients with suspected sepsis and respiratory distress. NIV is prioritized for patients with COPD or chronic heart failure presenting with one of the following [20, 21]: respiratory rate > 30 breaths/min, persistent hypoxemia despite oxygen therapy, pH 7.25–7.35, or PaCO2 > 45 mmHg, unless contraindicated by reduced consciousness, excessive secretions, or intolerance to NIV. In such cases, HFNC is used as an alternative.

NIV was delivered via an oronasal mask. The initial inspiratory positive airway pressure (IPAP) level was set at 10 to 15 cm H2O, and the expiratory positive airway pressure (EPAP) level was initially set at 5 to 6 cm H2O. The ventilator settings were adjusted depending on vital signs, blood gas data, and patient tolerance.

HFNC was delivered through large-bore nasal prongs. The initial flow rate was set at 40 to 60 L/min, and the FiO2 was set at 30–50%. The parameters were adjusted based on vital signs, blood gas data, and patient tolerance.

These settings represented the initial parameters. Patients in the NIV group requiring higher settings, such as increased PEEP levels to achieve alveolar recruitment, or patients in the HFNC group experiencing increased work of breathing requiring higher flow rates, could undergo subsequent adjustments as needed.

Patients undergoing these interventions are closely observed in the resuscitation area, continuously monitoring vital signs, follow-up blood gas levels, and assessments for potential intubation. Intubation is performed based on hemodynamic instability, airway compromise, excessive secretions, declining oxygenation, worsening hypercapnia despite NIV, and attending physician judgment.

### Data collection and study variables

We collected data on patient characteristics, including age, sex, body mass index (BMI), comorbid conditions, and vital signs at triage, such as heart rate, systolic blood pressure (SBP), mean arterial pressure (MAP), respiratory rate, oxygen saturation measured by pulse oximetry (SpO2), body temperature, and Glasgow Coma Scale (GCS) score. Biologic parameters were also recorded, including complete blood count, platelet count, creatinine, total bilirubin, serum bicarbonate, arterial pH, partial pressure of arterial oxygen (PaO2), partial pressure of arterial carbon dioxide (PaCO2), PaO2-to-FiO2 ratio (PF ratio), SpO2-to-FiO2 ratio (SF ratio), arterial lactate levels, and the site of infection. Additionally, we documented the initial settings of HFNC or NIV and the administration of vasoactive agents and corticosteroids.

The primary endpoint of the study was the proportion of patients requiring intubation within 48 h. Secondary outcomes included 28-day mortality (defined as all-cause mortality within 28 days of diagnosis), hospital length of stay, and ventilator-free days at day 28 (the number of days alive without the use of mechanical ventilation within 28 days of sepsis diagnosis).

### Sample size and statistical analysis

The sample size was calculated based on pilot data collected from sepsis patients in our department between June 1, 2022, and August 31, 2022. A total of 82 patients with suspected sepsis and acute respiratory distress who received either HFNC or NIV for respiratory support were included. The intubation rates for patients receiving HFNC and NIV were 25.81% (8 out of 31) and 15.69% (8 out of 51), respectively. Using Stata software for cohort studies, the sample size was calculated with P1 = 0.1569, P2 = 0.2581, a ratio of 1.6537, alpha (α) = 0.05, and beta (β) = 0.20. The required sample size was determined to be 544 patients, with 205 patients in the HFNC group and 339 in the NIV group.

Descriptive statistics were calculated for all clinical characteristics and relevant variables. Continuous data were presented as mean (standard deviation; SD) for normally distributed variables or as median (interquartile range; IQR) for non-normally distributed data. Comparisons were made using an independent t-test or Mann-Whitney U test, as appropriate. Categorical data were presented as percentages and compared using Fisher’s exact test.

Primary and secondary analyses, focusing on the incident of intubation and 28-day mortality, involved multivariable Cox regression to estimate hazard ratios (HR). Hospital length of stay and 28-day ventilator-free days were compared using multivariable Gaussian regression analysis for continuous outcomes, explicitly examining the mean differences between the HFNC and NIV groups. We used the DAGitty model to select the adjusted variables. Four factors, including Glasgow coma scale (GCS), respiratory tract infection, PaCO2, and PaO2-to-FiO2 ratio (PF ratio), were found to be minimally sufficient adjustment sets for estimating the overall effect of HFNC or NIV on intubation after 14 candidate covariate factors were input into the model (Supplement 1).

The Cox proportional hazards model was employed to analyze the cumulative incidence of intubation, and Kaplan-Meier curves were generated to assess the time from ED presentation to death. Comparisons were conducted using the log-rank test. All statistical tests were two-sided, with significance set at a P-value of less than 0.05. Data were analyzed using Stata version 16 (StataCorp LLC, College Station, TX, USA).

## Results

**Fig. 1:**
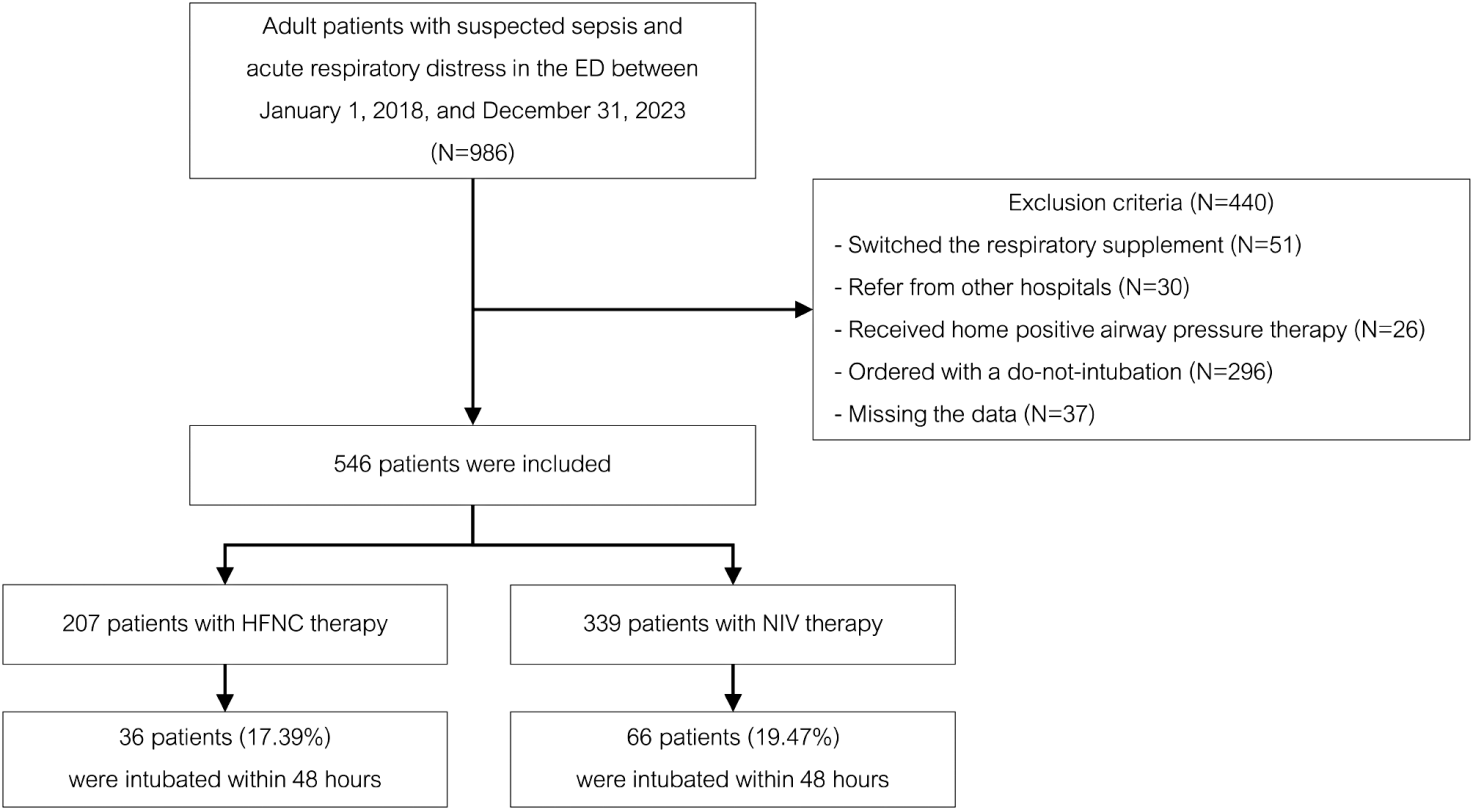
Study flow chart

A total of 986 patients with suspected sepsis and acute respiratory distress who received HFNC or NIV in the ED of Ramathibodi Hospital were initially identified. After excluding 440 patients—comprising 51 patients who switched respiratory modalities, 30 patients referred from other hospitals 26receiving home-positive airway pressure therapy, and 296 patients with do-not-intubate orders—546 were deemed eligible for inclusion in the study. Of these, 207 (37.91%) patients were in the HFNC group, and 339 (62.09%) patients were in the NIV group (as shown in Fig. 1: Study Flow).

### Patient characteristics

The demographic and clinical characteristics of patients are presented in Table 1. The overall mean age of the two groups was 74.69 years, with patients in the HFNC group being significantly older than those in the NIV group (76.69 ± 12.82 vs. 73.47 ± 14.08 years, *p* = 0.008). However, patients in the NIV group had a significantly higher mean BMI compared to the HFNC group (24.11 vs. 22.44, *p* < 0.001). The most common comorbidities observed in this study were hypertension (62.64%), dyslipidemia (46.89%), and diabetes (45.79%).

**Table 1:** Patients baseline characteristics and clinical outcome between HFNC and NIV treatment of suspected sepsis patients in emergency department

Variables	Total (*n* = 546)	HFNC (*n* = 207)	NIV (*n* = 339)	*P*-value
Age, mean (SD)	74.69 (13.69)	76.69 (12.82)	73.44 (14.09)	0.008
Male gender, *n* (%)	267 (48.90)	91 (43.96)	176 (51.92)	0.078
Body mass index, mean (SD)	23.48 (5.30)	22.44 (4.94)	24.11 (5.43)	< 0.001
Body mass index < 18, *n* (%)	74 (13.55)	38 (18.36)	36 (10.62)	0.014
Body mass index > 25, *n* (%)	180 (32.97)	56 (27.05)	124 (36.58)	0.024
**Comorbidities**, ***n*(%)**				
Hypertension	342 (62.64)	122 (58.94)	220 (64.90)	0.172
Diabetes Malleus	250 (45.79)	83 (40.58)	166 (48.97)	0.063
Dyslipidemia	256 (46.89)	95 (45.89)	161 (47.49)	0.725
Heart disease	212 (39.01)	65 (31.88)	147 (43.36)	0.009
Chronic heart failure	73 (13.37)	7 (3.38)	66 (19.47)	< 0.001
Chronic kidney disease	155 (28.39)	42 (20.29)	113 (33.33)	0.001
Vascular disease	54 (9.89)	19 (9.18)	35 (10.32)	0.768
Neurologic disease	175 (32.05)	84 (41.06)	90 (26.55)	< 0.001
COPD	85 (15.57)	24 (11.59)	61 (17.99)	0.052
Other respiratory tract disease	97 (17.77)	44 (21.26)	53 (15.63)	0.106
Liver disease	45 (8.24)	11 (5.31)	34 (10.03)	0.055
Cancer	124 (22.84)	51 (24.88)	73 (21.60)	0.464
Received immunosuppressive agent	57 (10.44)	25 (12.08)	32 (9.44)	0.387
HIV infection	9 (1.65)	2 (0.97)	7 (2.06)	0.494
**Clinical and biological parameters at inclusion**				
Heart rate (beats/min), mean (SD)	100.27 (22.04)	102.63 (20.90)	98.84 (22.61)	0.051
Systolic blood pressure (mmHg), mean (SD)	133.84 (29.18)	131.97 (26.80)	134.99 (30.51)	0.241
Mean arterial pressure (mmHg), mean (SD)	92.93 (18.07)	92.80 (17.19)	93.01 (18.62)	0.893
Respiratory rate (breaths/min), mean (SD)	27.54 (5.61)	27.57 (5.23)	27.52 (5.82)	0.913
SpO_2_ (%), mean (SD)	94.15 (5.56)	94.02 (5.02)	94.22 (5.88)	0.684
Body temperature (°C), mean (SD)	37.78 (1.08)	37.83 (1.10)	37.74 (1.06)	0.347
Glasgow Coma Scale (GCS), mean (SD)	14.25 (1.75)	13.82 (2.17)	14.52 (1.38)	< 0.001
WBC (*10^3^), median (IQR)	10.34 (7.51, 14.84)	10.78 (7.56, 15.05)	10.20 (7.40, 14.62)	0.684
Platelet (*10^3^), median (IQR)	209 (154, 281)	209 (154, 283)	208 (154, 279)	0.369
Creatinine (mg/dl), median (IQR)	1.11 (0.75, 1.80)	0.85 (0.67, 1.39)	1.28 (0.82, 2.02)	< 0.001
Bilirubin (mg/dl), median (IQR)	0.7 (0.5, 1.2)	0.7 (0.4, 1.3)	0.8 (0.5, 1.2)	0.145
Arterial pH, median (IQR) (*n* = 483)	7.43 (7.39, 7.47)	7.44 (7.40, 7.48)	7.43 (7.38, 7.46)	0.003
Arterial pH < 7.35, n (%) (*n* = 483)	29 (6.00)	7 (3.87)	22 (7.28)	0.166
Serum bicarbonate (mg/dl), mean (SD)	20.64 (4.45)	20.81 (3.94)	20.54 (4.73)	0.491
Serum lactate (mmol/L), median (IQR)	2.4 (1.7, 3.6)	2.4 (1.7, 3.6)	2.4 (1.7, 3.7)	0.873
PaO_2_, median (IQR) (*n* = 483)	108 (78, 165)	95 (72, 158)	115 (81, 170)	0.029
PaCO_2_, mean (SD) (*n* = 483)	34.60 (10.03)	33.76 (8.19)	35.09 (10.97)	0.160
PaCO_2_ > 45 mmHg, n (%) (*n* = 483)	56 (11.62)	17 (9.44)	39 (12.91)	0.304
PaO_2_-to-FiO_2_ ratio, mean (SD)	314.67 (133.48)	292.31 (129.22)	328.00 (134.41)	0.004
PaO_2_-to-FiO_2_ ratio ≤ 300, n (%)	235 (48.76)	96 (53.33)	139 (46.03)	0.132
SpO_2_-to-FiO_2_ ratio, mean (SD)	264.04 (92.31)	259.79 (92.76)	266.63 (92.07)	0.401
Bilateral infiltration, *n* (%)	146 (26.74)	64 (30.92)	82 (24.19)	0.091
**Site of infection**, ***n*(%)**				
Respiratory tract, *n* (%)	320 (58.61)	144 (69.57)	176 (51.92)	< 0.001
COVID-19 infection, *n* (%)	63 (11.54)	39 (18.84)	24 (7.08)	< 0.001
Gastrointestinal tract, *n* (%)	57 (10.44)	20 (9.66)	37 (10.91)	0.668
Urinary tract, *n* (%)	114 (20.88)	41 (19.81)	73 (21.53)	0.665
Skin and soft tissue, *n* (%)	17 (3.11)	2 (0.97)	15 (4.42)	0.023
Central nervous system, *n* (%)	5 (0.92)	4 (1.93)	1 (0.29)	0.071
Cardiovascular system, *n* (%)	1 (0.18)	0 (0)	1 (0.29)	1.000
CRBSI, *n* (%)	12 (2.20)	1 (0.48)	11 (3.24)	0.036
Primary bacteremia, *n* (%)	10 (1.83)	3 (1.45)	7 (2.06)	0.749
Unknown, *n* (%)	18 (3.30)	3 (1.45)	15 (4.42)	0.082
**Vasoactive agent**, ***n*(%)**				
One vasoactive agent, *n* (%)	88 (16.12)	35 (16.91)	53 (15.63)	0.720
> 1 vasoactive agent, *n* (%)	7 (1.28)	0	7 (2.06)	0.706
Received corticosteroid, *n* (%)	150 (27.47)	54 (26.09)	96 (28.32)	0.622
**Primary outcome**				
48-hour intubation, *n* (%)	102 (18.68)	36 (17.39)	66 (19.47)	0.573
- intubation at ED, *n* (%)	93 (17.03)	31 (5.68)	62 (11.36)	
- intubation at ICU, *n* (%)	9 (1.65)	5 (0.92)	4 (0.73)	
48-hour intubation in hypoxemia group^#^ (*n* = 280), *n* (%)	58 (20.71)	24 (20.51)	34 (20.86)	1.000
**Secondary outcome**				
28-day mortality, *n* (%)	23 (4.21)	6 (2.90)	17 (5.01)	0.277
28-day mortality in hypoxemia group^#^ (*n* = 280), n (%)	16 (5.71)	6 (5.13)	10 (6.13)	0.799
Length of stay (days), median (IQR)	8 (4, 15)	9 (4, 15)	8 (4, 15)	
Ventilator-free day at day 28 (days), mean (SD)	25.34 (6.67)	25.84 (5.73)	25.03 (7.17)	0.704

^#^hypoxemia group: defined as having a PaO_2_:FiO_2_ ratio of 300 mm Hg or less or a SpO_2_:FiO_2_ ratio of 315 or less

Abbreviations: HFNC, high-flow nasal cannula; NIV, noninvasive ventilation; COPD, chronic obstructive pulmonary disease; HIV infection, human immunodeficiency virus infection; SpO_2_, oxygen saturation; WBC, white blood cell count; PaO_2_, partial pressure of oxygen in the arterial blood; PaCO_2_, partial pressure of carbon dioxide in the arterial blood; FiO_2_, fraction of inspired oxygen; COVID-19 infection, infectious disease caused by the SARS-CoV-2 virus; CRBSI, catheter-related bloodstream infection; ED, emergency department; ICU, intensive care unit; SD, standard deviation; IQR, interquartile range.

The study included patients with various comorbidities: heart disease (43.36%), chronic heart failure (19.47%), chronic kidney disease (CKD) (33.33%), and chronic obstructive pulmonary disease (COPD) (17.99%), all of which were significantly associated with NIV use (*p* = 0.009, < 0.001, 0.001, and 0.052, respectively). In contrast, patients with neurological diseases (41.06%, *p* < 0.01) were more likely to receive respiratory support with HFNC.

Baseline vital signs did not differ significantly between the groups. However, the GCS score in the HFNC group was lower than in the NIV group (13.82 ± 2.17 vs. 14.52 ± 1.38, *p* < 0.001). The NIV group had a higher creatinine level than the HFNC group (1.28 vs. 0.85 mg/dL, respectively, *p* < 0.001). Additionally, the arterial pH was lower in the NIV group (pH 7.43, IQR 7.38–7.46) compared to the HFNC group (pH 7.44, IQR 7.40–7.48). Patients in the HFNC group had lower PaO2 levels (95, IQR 72–158) and a lower PF ratio (292.31 ± 129.22) compared to the NIV group (PaO2 115, IQR 81–170; PF ratio 328.00 ± 134.41; *p* = 0.029 and < 0.001, respectively). However, PaCO2 levels were not significantly different between the two groups (HFNC group 33.76 ± 8.19 vs. NIV group 35.09 ± 10.97; *p* = 0.160). Among the patients with hypercapnia (PaCO2 > 45 mmHg), 56 cases were identified from a total of 483 patients who underwent arterial blood gas analysis. The proportion was higher in the NIV group (39 cases, 12.91%) compared to the HFNC group (17 cases, 9.44%). However, the difference was not statistically significant (*p* = 0.304). Incidence of intubation within 48 h occurred in 1.83% (10 cases) and 0.92% (5 cases), respectively.

Among patients with respiratory tract infections, including COVID-19 infection, HFNC use was significantly more common, with 69.57% of patients receiving this modality (*p* < 0.001). Other sites of infection did not differ significantly between the groups, except for skin and soft tissue infection and catheter-related bloodstream infection, which were more prevalent in the NIV group (*p* = 0.023 and 0.036, respectively). The proportion of patients with septic shock, defined as those requiring vasoactive agents, did not differ significantly between the two groups.

### Respiratory support

Respiratory support with HFNC or NIV was initiated after the patient’s presentation to the ED, with a mean initiation time of approximately 2.58 h. For the HFNC group, the initial mean settings included a gas flow rate of 51.62 ± 7.07 L per minute and a mean FiO2 of 0.40 ± 0.09. In the NIV group, the initial settings comprised an inspiratory positive airway pressure (IPAP) level of 11.47 ± 2.81 cm H2O, an expiratory positive airway pressure (EPAP) level of 6.25 ± 0.96 cm H2O, and a mean FiO2 of 0.38 ± 0.09.

### Primary and secondary outcomes

**Fig. 2:**
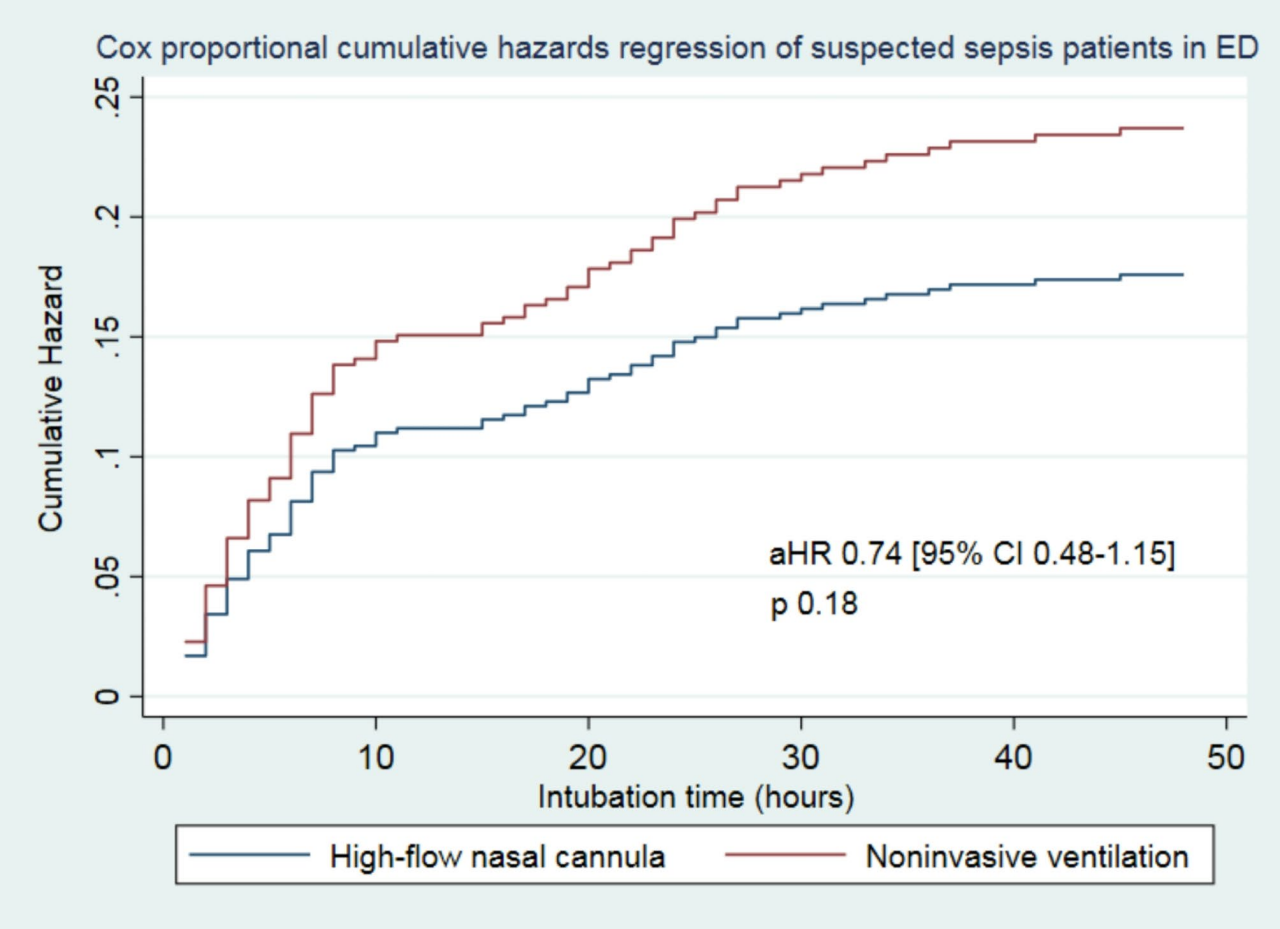
The Cox proportional hazards model was utilized to analyze the cumulative incidence of intubation from the time of emergency department presentation to 48 h. The model was adjusted for the Glasgow Coma Scale (GCS), PaCO2 levels, PaO2-to-FiO2 ratio, and the presence of respiratory tract infections

At 48 h, the overall incidence of intubation in the cohort was 18.68%, with 17.39% in the HFNC group and 19.47% in the NIV group (*p* = 0.573). In our study, the HFNC group exhibited a lower risk of intubation within 48 h compared to the NIV group; however, this difference was not statistically significant (crude hazard ratio [HR]: 0.88; 95% confidence interval [CI]: 0.59–1.32; *p* = 0.573). After adjusting for Glasgow Coma Scale (GCS), respiratory tract infection, PaCO2 levels, and PF ratio, the difference remained non-significant (adjusted hazard ratio [aHR]: 0.74; 95% CI: 0.48–1.15; *p* = 0.180), as shown in Table 2; Fig. 2.

**Table 2:** Univariable and multivariable regression analysis of clinical outcome between HFNC and NIV treatment of suspected sepsis patients in emergency department

Outcome High-flow nasal cannula vs. Noninvasive ventilation	Crude Hazard Ratio / Mean difference	*P*-value	Adjust Hazard Ratio / Mean difference	*P*-value
**Primary outcome**				
48-hour intubation (total *n* = 546)^A^	HR 0.88 [95% CI 0.59, 1.32]	0.573	aHR 0.74 ^a^ [95% CI 0.48, 1.15]	0.180
48-hour intubation in hypoxemia group^#^ (*n* = 280)^A^	HR 0.98 [95% CI 0.58, 1.65]	1.000	aHR 0.76 ^b^ [95% CI 0.43, 1.35]	0.350
**Secondary outcome**				
28-day mortality (total *n* = 546)^A^	HR 0.57 [95% CI 0.22, 1.44]	0.277	aHR 0.34 ^a^ [95% CI 0.12, 1.02]	0.053
28-day mortality in hypoxemia group^#^ (*n* = 280)^A^	HR 0.82 [95% CI 0.30, 2.26]	0.799	aHR 0.56 ^b^ [95% CI 0.17, 1.81]	0.329
Hospital length of stay (days)^B^	MD -0.07 [95% CI -2.24, 2.11]	0.704	AMD − 0.47 ^a^ [95% CI -2.98, 2.04]	0.713
Ventilator-free day at day 28 (days)^B^	MD 0.81 [95% CI -0.35, 1.96]	0.170	AMD 1.46 ^a^ [95% CI 0.11, 2.80]	0.034

a: adjust for Glasgow Coma Scale (GCS), PaCO_2_, PaO_2_-to-FiO_2_ ratio, and respiratory tract infection

b: adjust for Glasgow Coma Scale (GCS), PaCO_2_, and respiratory tract infection

^#^hypoxemia group: defined as having a PaO_2_:FiO_2_ ratio of 300 mm Hg or less or a SpO_2_:FiO_2_ ratio of 315 or less.

Abbreviations: HFNC, high-flow nasal cannula; NIV, noninvasive ventilation; vs., versus; HR, hazard ratio; aHR, adjust hazard ratio; MD, mean difference; AMD, adjusted mean differences; 95% CI, 95% confidence interval.

A: Univariable and multivariable Cox’s regression analysis

B: Univariable and multivariable Gaussian regression analysis

**Fig. 3:**
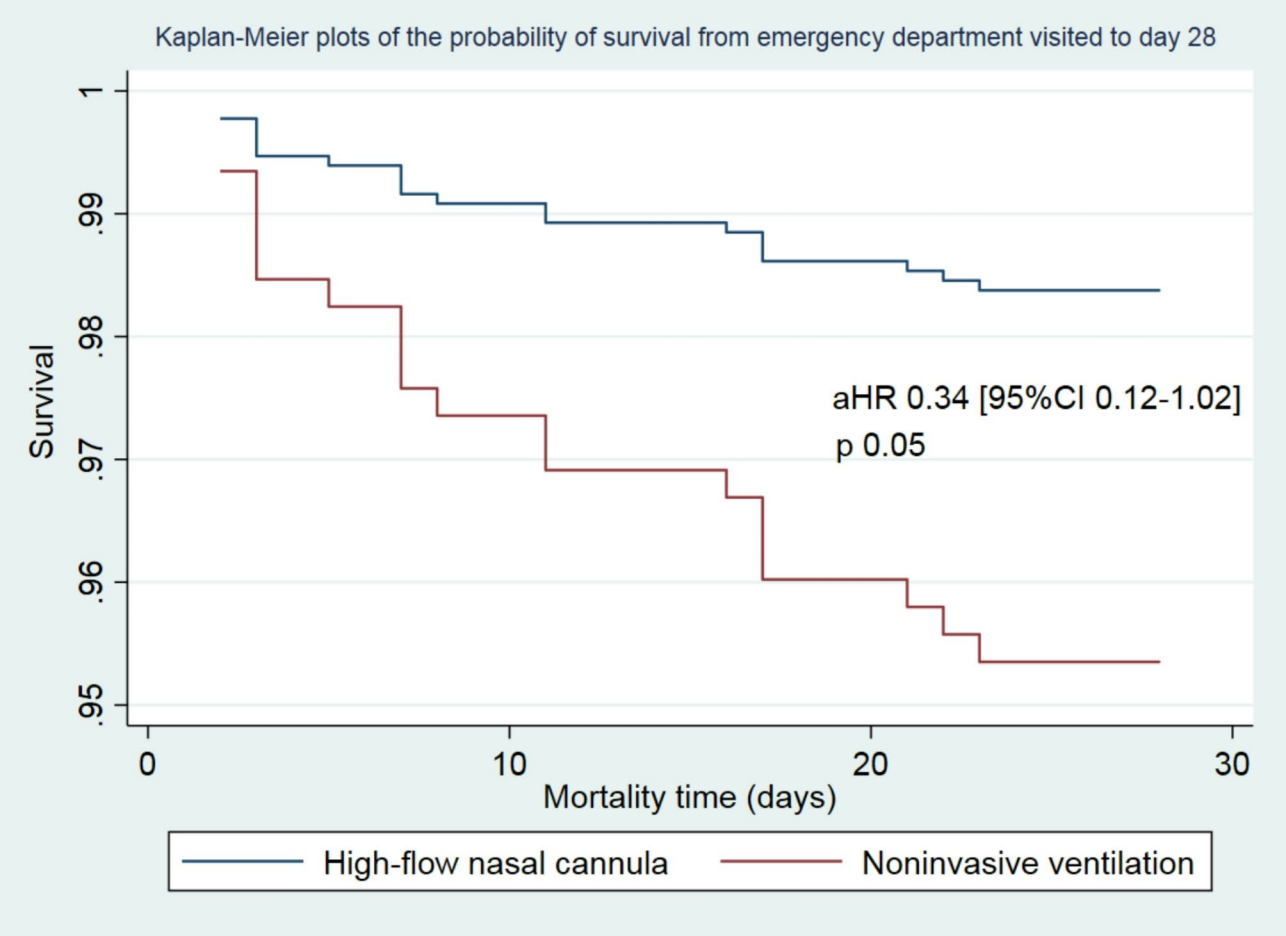
Kaplan-Meier survival curves were generated to illustrate the probability of survival from the time of emergency department presentation to day 28. The analysis was adjusted for key variables, including the Glasgow Coma Scale (GCS), PaCO2 levels, PaO2-to-FiO2 ratio, and the presence of respiratory tract infections

There was no significant difference in the 28-day mortality between the two groups (*p* = 0.277), with six patient deaths reported in the HFNC group and 17 in the NIV group. The hazard ratio (HR) for 28-day mortality when comparing HFNC to NIV was 0.57 (95% confidence interval [CI]: 0.22–1.44; *p* = 0.277). However, after adjusting for the Glasgow Coma Scale (GCS) score, respiratory tract infection, PaCO2 levels, and PF ratio, the risk of death at 28 days tended to be lower in the HFNC group, reaching marginal statistical significance (adjusted hazard ratio [aHR]: 0.34; 95% CI: 0.12–1.02; *p* = 0.053), as shown in Table 2; Fig. 3.

The median length of hospital stay was not significantly different between the two groups: 9 days in the HFNC group (interquartile range [IQR]: 4–15 days) and 8 days in the NIV group (IQR: 4–15 days). Although the HFNC group demonstrated a trend toward shorter hospital stays after adjustment for GCS score, respiratory tract infection, PaCO2 levels, and PF ratio, this difference was not statistically significant (adjust mean difference: -0.47 days; 95% CI: -2.98 to 2.04; *p* = 0.713), as shown in Table 2.

The number of ventilator-free days on day 28 also did not differ significantly between the groups (HFNC group: 25.84 ± 5.73 days; NIV group: 25.03 ± 7.17 days; *p* = 0.704). However, after adjustment for GCS, respiratory tract infection, PaCO2 levels, and PF ratio, the HFNC group had significantly more ventilator-free days than the NIV group, with adjust mean difference of 1.46 days (95% CI: 0.11 to 2.80; *p* = 0.034), as shown in Table 2.

The incidence of intubation among hypoxemic patients, defined as those with a PaO2:FiO2 ratio of 300 mm Hg or less or a SpO2:FiO2 ratio of 315 or less, was 20.51% in the HFNC group and 20.86% in the NIV group (*p* = 1.000). Although not statistically significant, the HFNC group demonstrated a trend toward a lower intubation rate within 48 h compared to the NIV group (hazard ratio [HR]: 0.98; 95% confidence interval [CI]: 0.58–1.65; *p* = 1.00). Adjustments for GCS score, respiratory tract infection, and PaCO2 levels did not substantially alter this trend (adjusted hazard ratio [aHR]: 0.76; 95% CI: 0.43–1.35; *p* = 0.350), as shown in Table 2.

Similarly, the 28-day mortality risk among hypoxemic patients was not significantly different between the HFNC and NIV groups. After adjusting for GCS, respiratory tract infection, and PaCO2 levels, the adjusted hazard ratio was 0.56 (95% CI: 0.17–1.81; *p* = 0.329), as shown in Table 2.

## Discussion

In this retrospective cohort study of patients with suspected sepsis and acute respiratory distress, initial respiratory support with HFNC compared to NIV in the ED did not result in significant differences in the incidence of 48-hour intubation, 28-day mortality, or hospital length of stay. However, the HFNC group exhibited a trend toward a reduced risk of 28-day mortality compared to the NIV group. Additionally, the HFNC group had significantly more ventilator-free days on day 28 than the NIV group.

Our study found that the 48-hour intubation threshold was correlated with the severity of the patient’s primary condition. Previous research has shown that delayed failure of HFNC or NIV—defined as failure occurring more than 48 h after the initiation of these therapies—is associated with poor outcomes [20–22]. These findings are consistent with earlier studies conducted in the ED, which reported no significant differences in intubation rates between patients with nonspecific respiratory failure supported by HFNC and those supported by NIV [16].

Furthermore, subgroup analyses in our study indicated no significant differences in the proportion of intubation between the HFNC and NIV groups. This result aligns with prior studies investigating patients with acute hypoxemic respiratory failure, which also reported no significant differences in intubation rates between these two respiratory support modalities [14, 15, 23, 24]. However, in contrast to our findings, a study by Tonelli et al. suggested that NIV is considerably less effective and less safe when used for patients with new-onset acute hypoxemic respiratory failure [25].

Recent guidelines recommend HFNC over NIV for patients with sepsis and respiratory failure [11]. However, several studies have reported no significant differences in intubation rates between the two modalities [14–16, 23, 24]. HFNC provides physiological benefits, enhances patient comfort, and is associated with fewer complications, making it particularly suitable for older patients and those with neurological comorbidities or respiratory tract infections [12, 26].

In our cohort, emergency physicians appeared to favor NIV for patients with sepsis who had comorbid conditions such as CKD, COPD, and heart failure. Despite this preference, our findings revealed no significant difference in intubation rates between the HFNC and NIV groups. Similarly, a recent randomized trial comparing HFNC and NIV in patients with acute exacerbation of COPD and hypercapnic respiratory failure demonstrated that HFNC is an effective treatment modality and enhances patient comfort [27]. However, we emphasize the importance of careful patient selection for NIV, strict adherence to established clinical guidelines, and close monitoring of patients undergoing NIV to minimize associated risks.

In this study, initial treatment with HFNC was associated with a reduction in 28-day mortality compared to NIV. This finding aligns with the study by Frat et al. [14], which reported a 2.5-fold higher hazard of death in patients treated with NIV. Furthermore, a recent systematic review and meta-analysis [15] identified a trend toward lower ICU mortality with HFNC, although the results did not reach statistical significance.

However, our findings contrast with those of Munroe et al. [24], a propensity score-matched study that found initial treatment with NIV to be associated with lower mortality compared to HFNC in patients presenting to the ED with acute hypoxemic respiratory failure. Notably, in that study, only 24% of the patient population had sepsis, which may account for the discrepancy, as our study specifically focused on sepsis-related acute respiratory distress.

The observed lower mortality in the HFNC group may be partially attributed to differences in baseline patient characteristics. The NIV group exhibited a higher prevalence of comorbidities, including chronic heart failure, CKD, and baseline metabolic acidosis, as indicated by elevated creatinine levels and lower arterial pH. These factors likely contributed to the poorer outcomes observed in the NIV group.

In contrast to the findings of Frat et al. [14], our study did not identify significant differences in mortality among patients with hypoxemia (PaO2-to-FiO2 ratio ≤ 300 mm Hg). This discrepancy may be explained by our cohort’s relatively less severe illness, as evidenced by higher baseline PaO2 levels, better PaO2-to-FiO2 ratios, and lower initial respiratory support settings. These differences in disease severity and treatment context emphasize the need for further research to identify optimal respiratory support strategies for specific subgroups of patients with sepsis and acute respiratory distress.

A key strength of our study is that it is the first to compare NIV and HFNC in patients with sepsis specifically. This is particularly important in the ED, where decisions to select either HFNC or NIV are often made prior to the establishment of a definitive diagnosis.

## Limitation

Our study has several limitations. First, the retrospective design, utilizing data from a single tertiary care center, may limit the generalizability of our findings to other settings. Second, we did not obtain a final diagnosis for all patients suspected of sepsis, meaning some patients may not have received a definitive diagnosis. Third, we did not collect data on hospital admission or ED disposition. However, all patients enrolled in the sepsis protocol received treatment according to the standard guidelines of Ramathibodi Hospital. Particularly, patients with shock or lactate levels > 4 mmol/L were managed in the resuscitation area with initial fluid resuscitation, early vasopressors, antibiotics, and consideration of systemic corticosteroids. However, there is no data on further ICU management, such as respiratory care, dexamethasone for ARDS, and urine output and fluid balance, which may have influenced the 28-day mortality rate. Fourth, we assessed 28-day all-cause mortality, which limits our ability to evaluate specific causes of death related to sepsis. Lastly, the data were collected from patients treated under the Ramathibodi sepsis protocol, which may have introduced selection or misclassification bias. Future randomized trials may provide more robust evidence on different outcomes.

## Conclusion

HFNC did not significantly reduce the number of intubation incidents. However, HFNC proved to be a beneficial strategy, associated with a lower mortality risk and a more significant number of ventilator-free days on day 28 compared to non-invasive ventilation (NIV) in patients with suspected sepsis and acute respiratory distress.

## Electronic supplementary material

Below is the link to the electronic supplementary material.

Supplement 1

Supplement 2

## Data Availability

The datasets used and/or analyzed during the current study are available from the corresponding author on reasonable request.

## Declarations

### Using artificial inteligence chatbots

During the preparation of this work the author(s) used ChatGPT4.0 in order to check and correct grammatical error during the manuscript writing process. After using this tool/service, the author(s) reviewed and edited the content as needed and take(s) full responsibility for the content of the publication.
