# Supplementary material for: Comparison of high-flow nasal oxygen therapy and noninvasive ventilation in suspected sepsis patients with acute respiratory distress in the emergency department: a retrospective cohort study
Source: Int J Emerg Med. 2025 Mar 10;18:52. doi: 10.1186/s12245-025-00842-2 (PMC11895199; doi:10.1186/s12245-025-00842-2)
Supplement: Supplementary file 1 — Supplement 1 [file 12245_2025_842_MOESM1_ESM.docx]

**Supplement 1**


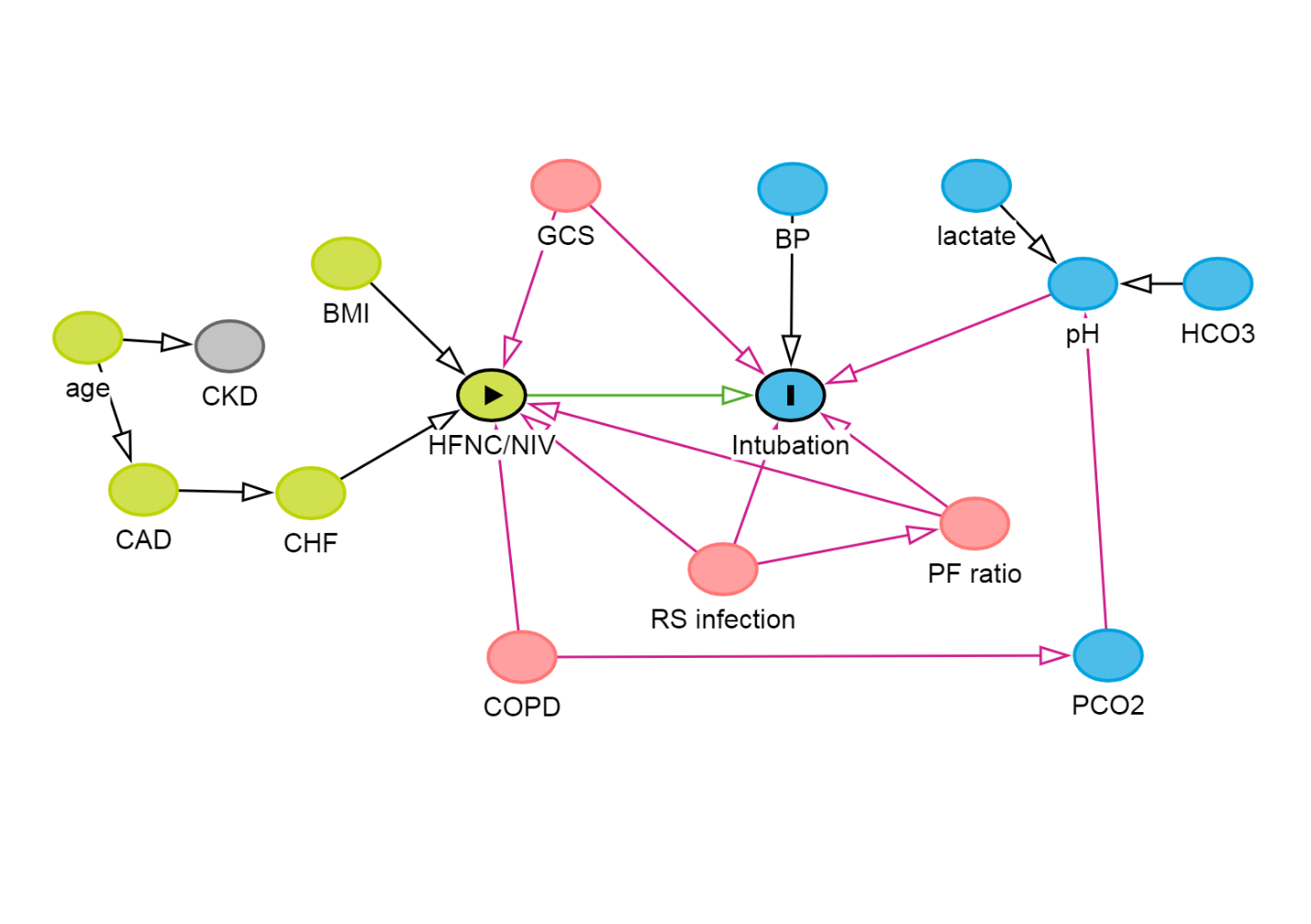


**Figure 1.** Illustration of the selection of adjustment variables for estimating the overall effect of HFNC or NIV on intubation using the DAGitty model.
