# Supplementary material for: Comparison of high-flow nasal oxygen therapy and noninvasive ventilation in suspected sepsis patients with acute respiratory distress in the emergency department: a retrospective cohort study
Source: Int J Emerg Med. 2025 Mar 10;18:52. doi: 10.1186/s12245-025-00842-2 (PMC11895199; doi:10.1186/s12245-025-00842-2)
Supplement: Supplementary file 2 — Supplement 2 [file 12245_2025_842_MOESM2_ESM.docx]

**Supplement 2**

**Ramathibodi sepsis protocol**

Ramathibodi Early Warning Score (REWS) was employed to predict the outcome of in-hospital mortality, according to Sutherasarn et al. (17) Therefore, we implemented the REWs score in the outpatient context to augment the early identification of sepsis patients. As a result, upon triage, every suspected sepsis patient who came to the Ramathibodi Hospital emergency department always had a REWS score. (Table 3)

Patients 18 years and older who visited the Ramathibodi Hospital ED with suspected sepsis and were screened using the Ramathibodi sepsis protocol from August 1, 2019. Doctors followed the Ramathibodi sepsis protocol: Ramathibodi Early Warning Score (REWs) ≥4 cooperate with suspicious infection from history or physical examination.

**Table.** Ramathibodi early warning score (REWs) clinical parameters and rubric scale for each parameter.

| **REWS** | | | | | | |
| --- | --- | --- | --- | --- | --- | --- |
| **Score** | **RR** | **SpO_2_** | **Temperature** | **SBP** | **HR** | **Mental status** |
| **3** | <10 | <84 | <33.9 | <89 | <39 |  |
| **2** |  | 85-89 | 34-34.9 |  |  |  |
| **1** |  | 90-92 | 35-35.9 | 90-99 | 40-49 |  |
| **0** | 11-20 | >93 | 36-37.9 | 100-199 | 50-99 | Alert |
| **1** | 21-30 |  | 38-38.9 |  | 100-109 | Response to voice |
| **2** | 31-35 |  | >39.0 | >200 | 110-129 | Response to pain |
| **3** | >36 |  |  |  | >130 | Unresponsive |

**Abbreviations**: RR, respiratory rate; SpO_2_, oxygen saturation; SBP, systolic blood pressure; HR, heart rate.
